# Benefit profile of anticoagulant therapy in sepsis: a nationwide multicentre registry in Japan

**DOI:** 10.1186/s13054-016-1415-1

**Published:** 2016-07-29

**Authors:** Kazuma Yamakawa, Yutaka Umemura, Mineji Hayakawa, Daisuke Kudo, Masamitsu Sanui, Hiroki Takahashi, Yoshiaki Yoshikawa, Toshimitsu Hamasaki, Satoshi Fujimi, for the Japan Septic Disseminated Intravascular Coagulation (J-Septic DIC) study group, Shinjiro Saito, Shigehiko Uchino, Yusuke Iizuka, Wataru Matsunaga, Kohei Takimoto, Toshihiko Mayumi, Hideaki Arai, Takeo Azuhata, Fumihito Ito, Shodai Yoshihiro, Hayakawa Katsura, Tsuyoshi Nakashima, Takayuki Ogura, Eiichiro Noda, Yoshihiko Nakamura, Ryosuke Sekine, Motohiro Sekinao, Keiko Ueno, Yuko Okuda, Masayuki Watanabe, Akihito Tampo, Nobuyuki Saito, Yuya Kitai, Iwao Kobayashi, Yutaka Kondo, Sho Nachi, Toru Miike, Hiroshi Takahashi, Shuhei Takauji, Kensuke Umakoshi, Takafumi Todaka, Hiroshi Kodaira, Kohkichi Andoh, Takehiko Kasai, Yoshiaki Iwashita, Masato Murata, Masahiro Yamane, Kazuhiro Shiga, Naoto Hori

**Affiliations:** 1 Department of Emergency and Critical Care, Osaka General Medical Center, 3-1-56 Bandai-Higashi, Sumiyoshi-ku, Osaka, 558-8558 Japan; 2 Department of Traumatology and Acute Critical Medicine, Osaka University Graduate School of Medicine, 2-15 Yamadaoka, Suita, Osaka 565-0871 Japan; 3 Emergency and Critical Care Center, Hokkaido University Hospital, Kita 15 Nishi 7 Kita-ku, Sapporo, Hokkaido 060-8638 Japan; 4 Division of Emergency and Critical Care Medicine, Tohoku University Graduate School of Medicine, 2-1 Seiryo-machi, Aoba-ku, Sendai, Miyagi 980-8575 Japan; 5 Department of Anesthesiology and Critical Care Medicine, Jichi Medical University Saitama Medical Center, 1-847 Amanuma-cho, Omiya-ku, Saitama, 330-8503 Japan; 6 Office of Biostatistics and Data Management, National Cerebral and Cardiovascular Center, 5-7-1 Fujishirodai, Suita, Osaka 565-8565 Japan

**Keywords:** Anticoagulants, Sepsis, Retrospective studies, Subgroup analysis, Disseminated intravascular coagulation, DIC

## Abstract

**Background:**

Little evidence supports anticoagulant therapy as effective adjuvant therapy to reduce mortality overall in sepsis. However, several studies suggest that anticoagulant therapy may reduce mortality in specific patients. This study aimed to identify a subset of patients with high benefit profiles for anticoagulant therapy against sepsis.

**Methods:**

This post hoc subgroup analysis of a nationwide multicentre retrospective registry was conducted in 42 intensive care units in Japan. Consecutive adult patients with sepsis were included. Treatment effects of anticoagulants, e.g. antithrombin, recombinant thrombomodulin, heparin, and protease inhibitors, were evaluated by stratifying patients according to disseminated intravascular coagulation (DIC) and Sequential Organ Failure Assessment (SOFA) score. Intervention effects of anticoagulant therapy on in-hospital mortality and bleeding complications were analysed using Cox regression analysis stratified by propensity scores.

**Results:**

Participants comprised 2663 consecutive patients with sepsis; 1247 patients received anticoagulants and 1416 received none. After adjustment for imbalances, anticoagulant administration was significantly associated with reduced mortality only in subsets of patients diagnosed with DIC, whereas similar mortality rates were observed in non-DIC subsets with anticoagulant therapy. Favourable associations between anticoagulant therapy and mortality were observed only in the high-risk subset (SOFA score 13–17; adjusted hazard ratio 0.601; 95 % confidence interval 0.451, 0.800) but not in the subsets of patients with sepsis with low to moderate risk. Although the differences were not statistically significant, there was a consistent tendency towards an increase in bleeding-related transfusions in all SOFA score subsets.

**Conclusions:**

The analysis of this large database indicates anticoagulant therapy may be associated with a survival benefit in patients with sepsis-induced coagulopathy and/or very severe disease.

**Trial registration:**

University Hospital Medical Information Network Clinical Trial Registry (UMIN-CTR ID: UMIN000012543). Registered on 10 December 2013.

**Electronic supplementary material:**

The online version of this article (doi:10.1186/s13054-016-1415-1) contains supplementary material, which is available to authorized users.

## Background

Sepsis invariably leads to haemostatic abnormalities through the activation of inflammatory mediators and vascular endothelial cell injury, which play a critical role in inducing multiple organ dysfunction syndrome and subsequent death [1–4]. Thus, anticoagulant therapies are expected to be beneficial in the treatment of sepsis [5, 6]. Several anticoagulant therapies, such as recombinant human activated protein C, antithrombin, recombinant tissue factor pathway inhibitor, and recombinant human soluble thrombomodulin (rhTM), have already been evaluated as adjuvant therapy against sepsis [7–13]. However, the efficacy of anticoagulant therapies in sepsis remains a matter of dispute because of limited evidence that it improves clinical outcomes.

Although most previous studies of anticoagulants did not observe reduced mortality overall in patients with sepsis, some subgroup analyses suggest that anticoagulant therapies might be beneficial only in specific patients with sepsis. For example, the post hoc subgroup analysis of a large multinational randomised controlled trial (RCT) of antithrombin suggested that favourable treatment effects of antithrombin were observed only in patients with sepsis with predicted mortality between 30 % and 60 % according to the baseline Simplified Acute Physiology Score II score [14]. Additionally, antithrombin treatment was associated with a significant reduction in mortality only in patients suffering from sepsis-induced disseminated intravascular coagulation (DIC) and not in non-DIC patients [15]. We previously demonstrated significant association between recombinant human soluble thrombomodulin (rhTM) treatment and favourable mortality outcome only in patients with both sepsis-induced DIC and high risk of death according to baseline Acute Physiology and Chronic Health Evaluation (APACHE) II and Sequential Organ Failure Assessment (SOFA) scores [16].

We thus hypothesised that anticoagulant therapies might be effective only in patients with sepsis who are at high risk of death and have DIC, and might not be effective in other patients. This study aimed to analyse the association between anticoagulant therapy and outcomes in sepsis according to baseline DIC status and baseline disease severity.

## Methods

### Study population

This investigation was a post hoc subgroup analysis of a multicentre nationwide retrospective cohort study (the Japan Septic Disseminated Intravascular Coagulation (J-Septic DIC) registry) conducted in 42 intensive care units (ICUs) in Japan between January 2011 and December 2013 [17]. Patients were eligible for the registry if they had a known or suspected infection on the basis of clinical data and met the following criteria at the time of ICU admission: three or more signs of systemic inflammation and sepsis-induced dysfunction of at least one organ or system, and age ≥18 years. The exclusion criteria included the use of warfarin/acetylsalicylic acid/thrombolytic therapy before study entry; history of fulminant hepatitis, decompensated liver cirrhosis, or other serious liver disorder; history of haematologic malignant disease; other conditions increasing the risk of bleeding; treatment with any chemotherapy at study entry; and patients with missing data for primary evaluation.

Participants were categorised into one of two groups: the anticoagulant group, comprising patients who underwent systemic administration at therapeutic doses of any anticoagulant such as antithrombin, rhTM, heparin/heparinoid or serine protease inhibitors, and the control group comprising patients who received no anticoagulant therapy. There was no predefined protocol on definite indications for anticoagulant therapy. Anticoagulant therapy in patients with sepsis fulfilling the criteria for DIC was applied at the discretion of the attending physician based on the treatment principles of each hospital. The standard dosage and duration of treatment for each anticoagulant agent used for sepsis-induced DIC in Japan are shown in Additional file 1: Table S1. No patients were administered recombinant human activated protein C because it had not been approved for the treatment of sepsis in Japan. Patients receiving prophylactic administration of low-dose heparin/heparinoid for venous thromboembolism were included in both groups.

This study followed the principles of the Declaration of Helsinki and was approved by the institutional review board of each participating hospital (Additional file 1: Table S2). Because of the anonymous and retrospective nature of this study, the board of each hospital waived the need for informed consent. This study was registered with the University Hospital Medical Information Network Clinical Trial Registry (UMIN-CTR ID: UMIN000012543).

### Data collection

A case report form was developed on which the following information was recorded: age, sex, multiple illness severity scores on the day of ICU admission, source of ICU admission, pre-existing conditions, new organ dysfunction, primary source of infection, and concomitant therapies against sepsis. The severity of illness was evaluated at the time of ICU admission according to the APACHE II score and systemic inflammatory response syndrome score. Organ dysfunction, defined as a SOFA sub-score ≥2 for each organ, was assessed according to the SOFA score at the time of ICU admission. DIC was diagnosed on the basis of the criteria of the International Society on Thrombosis and Haemostasis (ISTH) and the Japanese Association for Acute Medicine (JAAM) at the time of ICU admission. Details of the scoring systems for the ISTH overt DIC [18, 19] and the JAAM DIC definitions [20] are summarised in Additional file 1: Table S3.

The primary outcome measure was all-cause in-hospital mortality. We also recorded as secondary outcomes any bleeding complications, including the occurrence of intracranial haemorrhage, transfusion requirements related to bleeding, and bleeding requiring surgical intervention.

### Statistical analysis

The aim of this study was to identify a subset of patients with high benefit profiles for anticoagulant therapy against sepsis. Classification and regression trees for survival data (survival CART) were thus used to classify patients according to disease severity as determined by SOFA and APACHE II scores and age.

Due to the retrospective nature of the study, there were baseline imbalances between the two patient groups; therefore, an adjusted mortality analysis was performed using propensity scores [21, 22]. The propensity score for receiving anticoagulant therapy was calculated using multivariate logistic regression and included 32 independent variables comprising age, sex, disease severity, source of ICU admission, past medical history of severe conditions, new organ dysfunction, ICU characteristics, primary source of infection, causal microorganisms, anticoagulant therapy for indications other than DIC, and other therapeutic interventions (see Additional file 1: Table S4). The *c* statistic was 0.818. The Hosmer-Lemeshow chi-square value was 12.840 (*df* = 8), with a nonsignificant *p* value of 0.117, which indicates that the model fit well.

Patients were stratified into quintiles according to their propensity scores. The overall association between treatment and mortality outcomes was assessed using a Cox regression model with strata defined by propensity score hazard ratio (HR) and estimated 95 % confidence interval (CI). For secondary outcomes of bleeding complications, the odds ratio (OR) and associated 95 % CI were estimated by logistic regression stratified by propensity score. Inverse probability-of-treatment weighting using the propensity score was also used to assess the robustness of the conclusions from the adjusted method, and no major significant differences between the methods were found.

Descriptive statistics were calculated as medians (interquartile range) or proportions, as appropriate. Univariate differences between groups were assessed using the Mann-Whitney *U* test, Kruskal-Wallis test, chi-square test, or Fisher’s exact test. A *p* value <0.05 indicated statistical significance. All statistical analyses were performed with IBM SPSS Statistics version 22.0 for Windows (SPSS Inc., Chicago, IL, USA), or R software package version 3.2.0 (R Development Core Team).

## Results

### Study population and stratification by survival CART

**Fig. 1:**
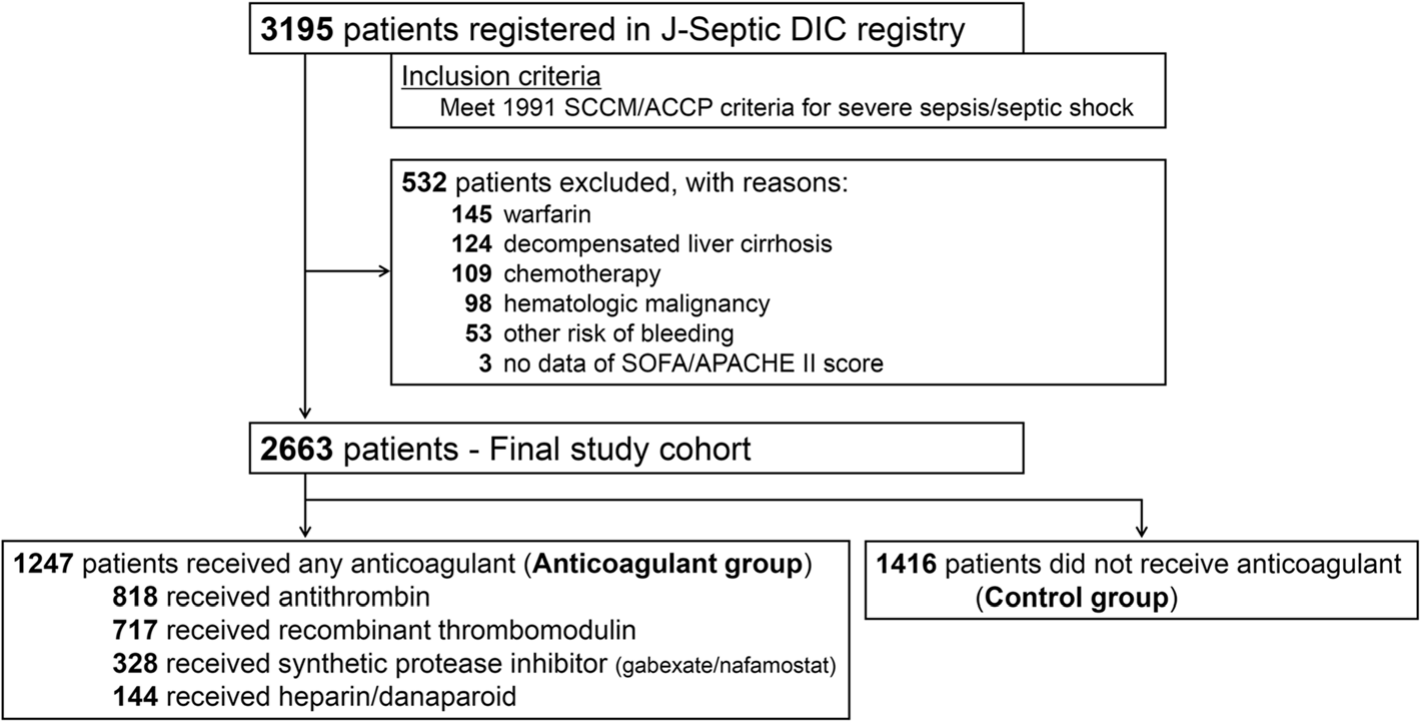
Patient flow diagram. *J-Septic DIC* Japan Septic Disseminated Intravascular Coagulation, *SCCM*/*ACCP* Society of Critical Care Medicine/American College of Chest Physicians, *SOFA* Sequential Organ Failure Assessment, *APACHE* Acute Physiology and Chronic Health Evaluation

The patient flow diagram is shown in Fig. 1. During the study period, 3195 consecutive patients fulfilling the inclusion criteria were registered in the J-Septic DIC registry database. After excluding 532 patients who met at least one exclusion criterion, we analysed 2663 patients as the final study cohort. The anticoagulant group comprised 1247 patients and the control group comprised 1416 patients.

**Fig. 2:**
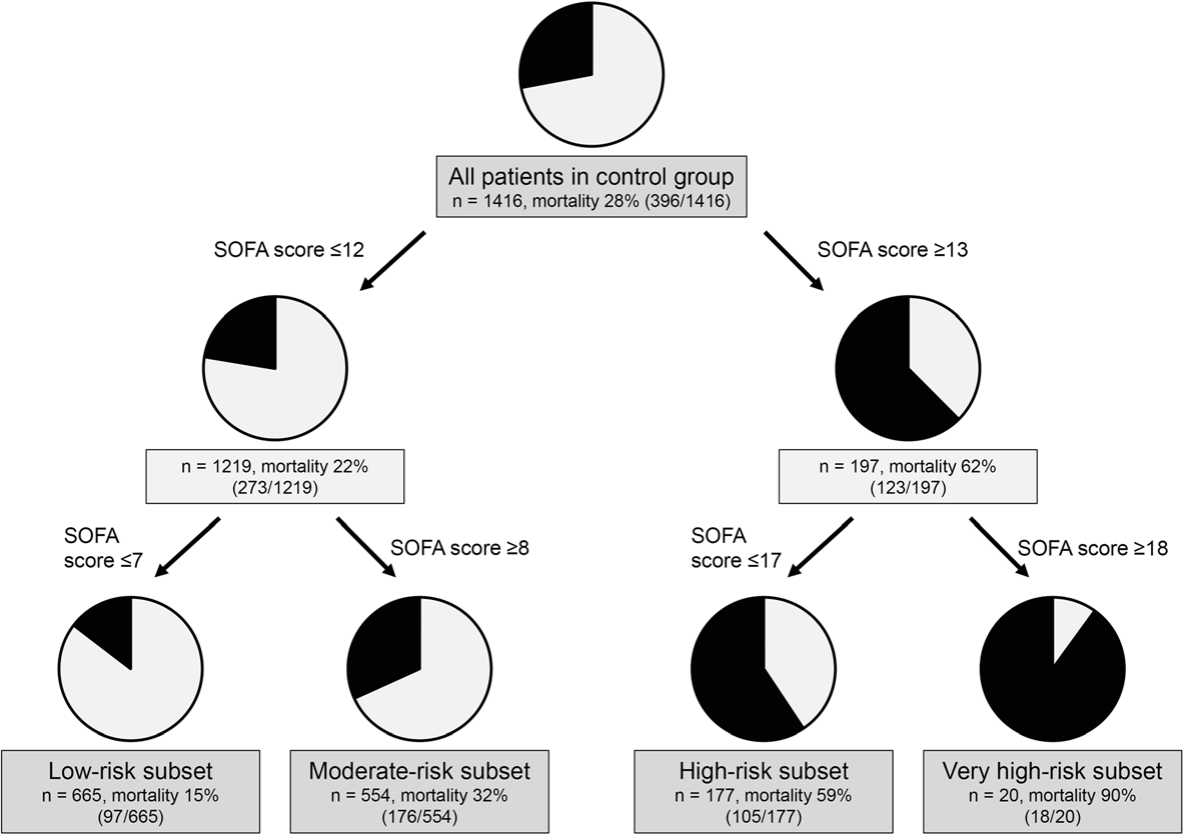
Patient stratification according to baseline Sequential Organ Failure Assessment (*SOFA*) score using the classification and regression tree method

Survival CART analysis of SOFA scores revealed that the first split point at which to partition mortality risk for patients without anticoagulant therapy was a SOFA score of 13, and the second split points were SOFA scores of 8 and 18 for all subsets of patients (Fig. 2). Therefore, the associations between anticoagulant therapy and outcomes were estimated in these four subsets. Patients were also classified in the same manner according to APACHE II score and age.

### Baseline characteristics

Baseline characteristics and therapeutic interventions of the overall study population are shown in Table 1. Although patient characteristics such as age and sex were similar between the anticoagulant and control groups, illness severity, as indicated by SOFA, APACHE II, and DIC scores, and the rate of new organ dysfunction showed significant differences between groups. The most common sites of underlying infection among all patients were the abdomen (33 %) and the lung (25 %), but these rates were not similar between treatment groups. The rate of concomitant therapeutic interventions against sepsis was also different between groups.

**Table 1:** Baseline characteristics of all patients treated or not treated with anticoagulants

	Overall (n = 2663)	Anticoagulant group (n = 1247)	Control group (n = 1416)	*P* value
Patient characteristics				
Age in years	73 (63–81)	72 (62–80)	73 (63–81)	0.034
Male sex	1576 (59 %)	716 (57 %)	860 (61 %)	0.089
Illness severity				
SIRS score	3 (2–4)	3 (2–4)	3 (2–4)	0.508
SOFA score	9 (6–12)	10 (8–13)	8 (5–11)	<0.001
APACHE II score	22 (16–28)	23 (18–29)	21 (16–27)	<0.001
ISTH DIC score	3 (1–4)	4 (2–5)	2 (1–4)	<0.001
JAAM DIC score	4 (2–6)	5 (3–6)	3 (2–5)	<0.001
Source of ICU admission				<0.001
Emergency department	1256 (47 %)	527 (42 %)	729 (52 %)	-
Ward	711 (27 %)	366 (29 %)	345 (24 %)	-
Other hospital	696 (26 %)	354 (28 %)	342 (24 %)	-
Pre-existing condition				
Liver insufficiency	19 (1 %)	12 (1 %)	7 (1 %)	0.171
Chronic heart failure	143 (5 %)	76 (6 %)	67 (5 %)	0.122
Chronic respiratory disorder	102 (4 %)	42 (3 %)	60 (4 %)	0.266
Chronic haemodialysis	209 (8 %)	84 (7 %)	125 (9 %)	0.051
Immunocompromised	280 (11 %)	130 (10 %)	150 (11 %)	0.899
New organ dysfunction (SOFA sub-scores ≥2)				
Respiratory	1792 (88 %)	851 (68 %)	941 (67 %)	0.341
Cardiovascular	1761 (66 %)	934 (75 %)	827 (58 %)	<0.001
Renal	1279 (48 %)	707 (57 %)	572 (40 %)	<0.001
Hepatic	441 (17 %)	253 (20 %)	188 (13 %)	<0.001
Coagulation	957 (36 %)	591 (47 %)	366 (26 %)	<0.001
Primary source of infection				<0.001
Abdomen	881 (33 %)	460 (37 %)	421 (30 %)	-
Lung	677 (25 %)	269 (22 %)	408 (29 %)	-
Urinary tract	456 (17 %)	221 (18 %)	235 (17 %)	-
Bone/soft tissue	309 (12 %)	146 (12 %)	163 (12 %)	-
Central nervous system	57 (2 %)	30 (2 %)	27 (2 %)	-
Other/unknown	283 (11 %)	121 (10 %)	162 (11 %)	-
Other therapeutic interventions				
Immunoglobulin	800 (30 %)	590 (47 %)	210 (15 %)	<0.001
Low-dose steroid	624 (23 %)	401 (32 %)	223 (16 %)	<0.001
Renal replacement therapy	830 (31 %)	566 (45 %)	264 (19 %)	<0.001
PMX-DHP	574 (22 %)	410 (33 %)	164 (12 %)	<0.001
Surgical intervention	1153 (43 %)	617 (50 %)	536 (38 %)	<0.001

Data are expressed as group medians (interquartile range) or proportion (%). *SIRS* Systemic Inflammatory Response Syndrome, *SOFA* Sequential Organ Failure Assessment, *APACHE* Acute Physiology and Chronic Health Evaluation, *ISTH* International Society on Thrombosis and Haemostasis, *DIC* disseminated intravascular coagulation, *JAAM* Japanese Association for Acute Medicine, *ICU* intensive care unit, *PMX-DHP* polymyxin B direct haemoperfusion

Additionally, baseline characteristics and therapeutic interventions in patients treated or not treated with anticoagulant in the specific subset according to baseline DIC status and SOFA score are shown in Table 2 and Additional file 1: Table S5, respectively. The anticoagulant and control groups of the DIC-positive subset were well balanced in age, sex, rate of new organ dysfunction, and primary source of infection, whereas in the DIC-negative subset, there were some differences between the two groups. Baseline severity of the coagulation disorder determined by JAAM DIC scores and the rate of concomitant therapeutic interventions were both significantly higher in the anticoagulant group relative to the control group in the two subsets with and without ISTH overt DIC.

**Table 2:** Baseline characteristics of the patients with and without DIC diagnosed by ISTH overt DIC criteria treated or untreated with anticoagulants

	Non-DIC (*n* = 2037)			DIC (*n* = 626)		
	Anticoagulant group (n = 814)	Control group (n = 1223)	*P* value	Anticoagulant group (n = 433)	Control group (n = 193)	*P* value
Patient characteristics						
Age in years	72 (63–80)	73 (63–81)	0.127	72 (61–80)	73 (63–82)	0.314
Male sex	485 (60 %)	743 (61 %)	0.611	231 (53 %)	117 (61 %)	0.098
Illness severity						
SIRS score	3 (2–4)	3 (2–4)	0.522	3 (3–4)	3 (3–4)	0.078
SOFA score	9 (7–12)	7 (5–10)	<0.001	12 (9–14)	12 (9–15)	0.520
APACHE II score	23 (17–28)	20 (15–26)	<0.001	25 (19–30)	24 (18–33)	0.342
ISTH DIC score	3 (2–4)	2 (1–3)	<0.001	6 (5–6)	6 (5–6)	0.411
JAAM DIC score	4 (3–5)	2 (1–4)	<0.001	7 (6–8)	6 (5–8)	0.004
Source of ICU admission			<0.001			0.395
Emergency department	349 (43 %)	639 (52 %)	-	178 (41 %)	90 (47 %)	-
Ward	233 (29 %)	294 (24 %)	-	133 (31 %)	51 (26 %)	-
Other hospital	232 (29 %)	290 (24 %)	-	122 (30 %)	52 (27 %)	-
Pre-existing condition						
Liver insufficiency	7 (0.9 %)	3 (0.2 %)	0.100	5 (1 %)	4 (2 %)	0.468
Chronic heart failure	49 (6 %)	60 (5 %)	0.315	27 (6 %)	7 (4 %)	0.251
Chronic respiratory disorder	31 (4 %)	50 (4 %)	0.817	11 (3 %)	10 (5 %)	0.097
Chronic haemodialysis	54 (7 %)	98 (8 %)	0.264	30 (7 %)	27 (14 %)	0.006
Immunocompromised	86 (11 %)	134 (11 %)	0.827	44 (10 %)	16 (8 %)	0.557
New organ dysfunction (SOFA sub-scores ≥2)						
Respiratory	565 (69 %)	812 (66 %)	0.161	286 (66 %)	129 (67 %)	0.927
Cardiovascular	605 (74 %)	681 (56 %)	<0.001	329 (76 %)	146 (76 %)	0.920
Renal	416 (51 %)	454 (37 %)	<0.001	291 (67 %)	118 (61 %)	0.147
Hepatic	114 (14 %)	136 (11 %)	0.054	139 (32 %)	52 (27 %)	0.222
Coagulation	239 (29 %)	226 (19 %)	<0.001	352 (81 %)	140 (73 %)	0.015
Primary source of infection			<0.001			0.257
Abdomen	304 (37 %)	342 (28 %)	-	156 (36 %)	79 (41 %)	-
Lung	205 (25 %)	383 (31 %)	-	64 (15 %)	25 (13 %)	-
Urinary tract	113 (14 %)	199 (16 %)	-	108 (25 %)	36 (19 %)	-
Bone/soft tissue	102 (13 %)	145 (12 %)	-	44 (10 %)	18 (9 %)	-
Central nervous system	15 (2 %)	22 (2 %)	-	15 (4 %)	5 (3 %)	-
Other/unknown	75 (9 %)	132 (11 %)	-	46 (11 %)	30 (16 %)	-
Other therapeutic interventions						
Immunoglobulin	377 (46 %)	179 (15 %)	<0.001	213 (49 %)	31 (16 %)	<0.001
Low-dose steroid	248 (31 %)	184 (15 %)	<0.001	153 (35 %)	39 (20 %)	<0.001
Renal replacement therapy	351 (43 %)	204 (17 %)	<0.001	215 (50 %)	60 (31 %)	<0.001
PMX-DHP	251 (31 %)	137 (11 %)	<0.001	159 (37 %)	27 (14 %)	<0.001
Surgical intervention	415 (51 %)	458 (37 %)	<0.001	202 (47 %)	78 (40 %)	0.164

Data are expressed as group medians (interquartile range) or proportion (%). *DIC* disseminated intravascular coagulation, *ISTH* International Society on Thrombosis and Haemostasis, *SIRS* Systemic Inflammatory Response Syndrome, *SOFA* Sequential Organ Failure Assessment, *APACHE* Acute Physiology and Chronic Health Evaluation, *JAAM* Japanese Association for Acute Medicine, *ICU* intensive care unit, *PMX-DHP* polymyxin B direct haemoperfusion

### Mortality according to baseline DIC status

**Fig. 3:**
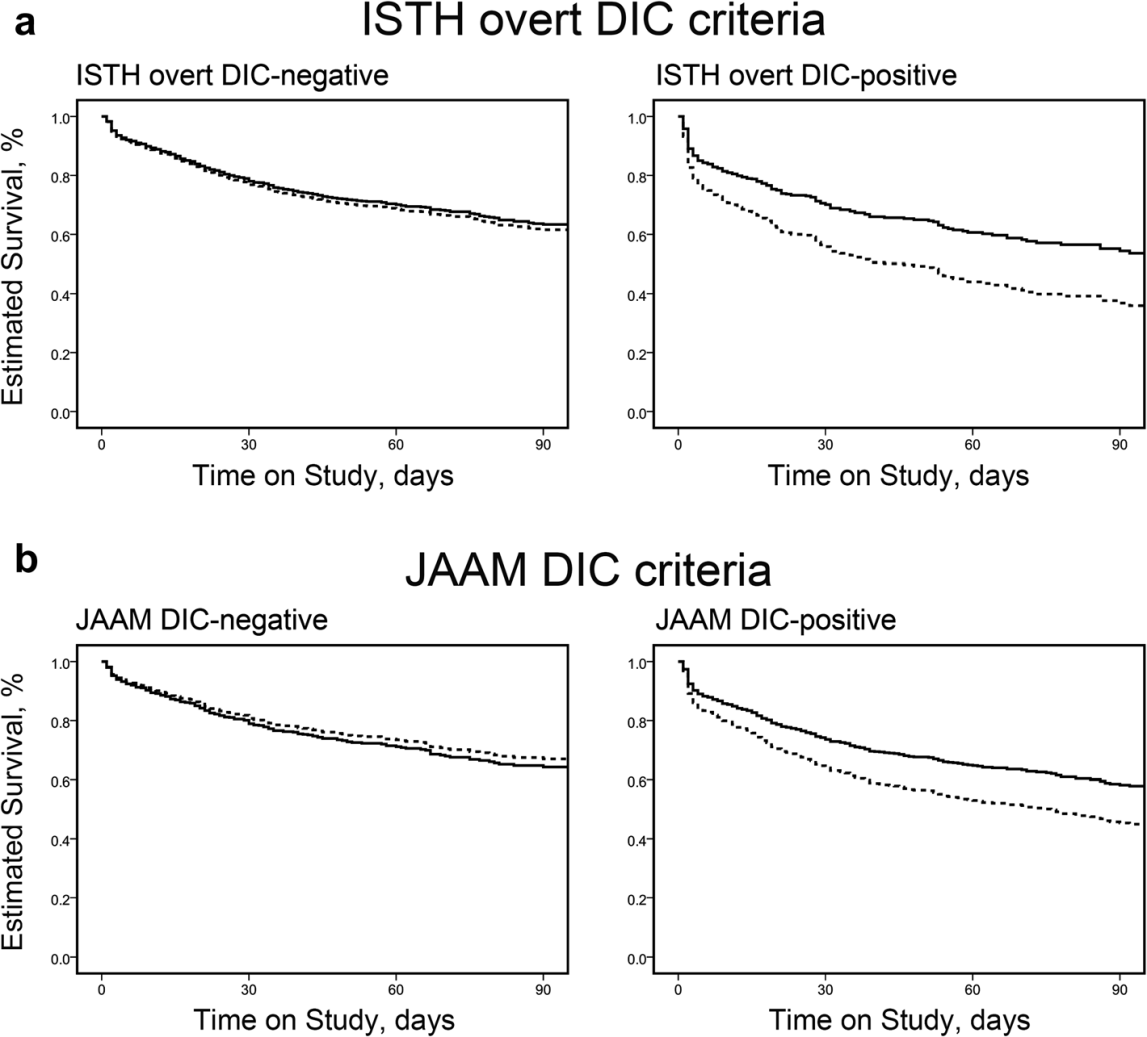
Adjusted estimated survival curves in patients with or without disseminated intravascular coagulation (*DIC*) diagnosed by ISTH overt DIC criteria (**a**) and JAAM DIC criteria (**b**). The *solid line* represents patients in the anticoagulant group, and the *dotted line* represents patients in the control group. *ISTH* International Society on Thrombosis and Haemostasis, *JAAM* Japanese Association for Acute Medicine

Survival curves for the anticoagulant and control groups in the prediction model are shown in Fig. 3, according to covariates of propensity scores for subsets determined by DIC status diagnosed using both the ISTH overt DIC and JAAM DIC criteria. Significant associations between anticoagulant therapy and lower in-hospital mortality were observed only in the subsets of patients diagnosed with DIC (adjusted HR 0.609; 95 % CI 0.456, 0.814; *p* = 0.001 for the subset positive for ISTH overt DIC and adjusted HR 0.685; 95 % CI 0.559, 0.839; *p* <0.001 for the subset positive for JAAM DIC), whereas similar mortality rates were observed in the non-DIC subsets with anticoagulant therapy (adjusted HR 0.941; 95 % CI 0.773, 1.145; *p* = 0.543 for the subset negative for ISTH overt DIC and adjusted HR 1.104; 95 % CI 0.839, 1.453; *p* = 0.478 for the subset negative for JAAM DIC).

### Mortality according to baseline SOFA score

**Fig. 4:**
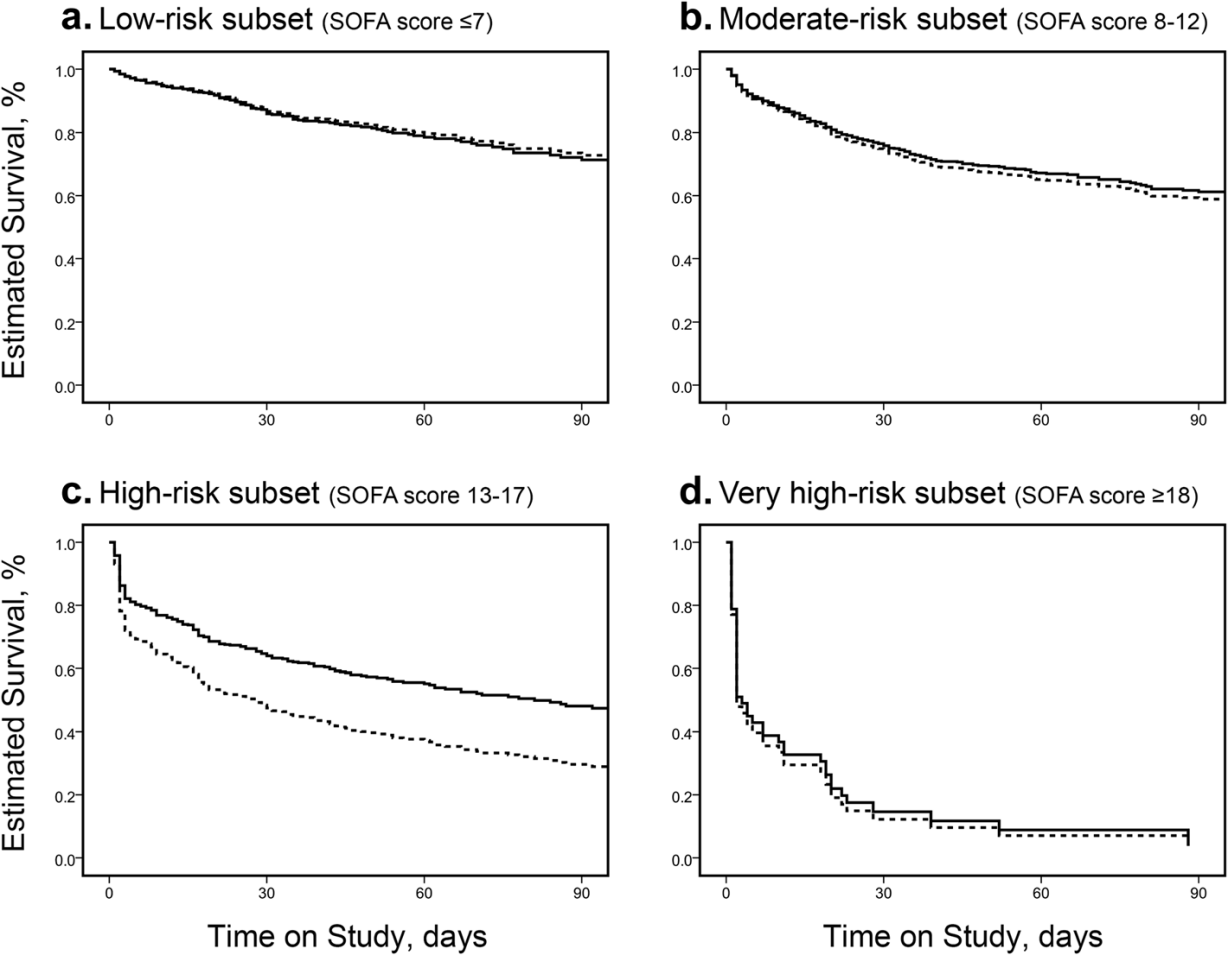
Adjusted estimated survival curves in 4 subsets stratified according to baseline Sequential Organ Failure Assessment (*SOFA*) score (**a**-**d**). The *solid line* represents patients in the anticoagulant group, and the *dotted line* represents patients in the control group

Survival curves for the anticoagulant and control groups in the prediction model are shown in Fig. 4 according to covariates of propensity scores for subsets determined by baseline SOFA scores. Cox regression analysis suggested that anticoagulant therapy was significantly associated with reduced mortality but only in patients in the high-risk subset (SOFA 13–17; adjusted HR 0.601; 95 % CI 0.451, 0.800; *p* <0.001). In contrast, no association with survival was evident in the low-risk subset (SOFA ≤7; adjusted HR 1.063; 95 % CI 0.716, 1.580; *p* = 0.761) and moderate-risk subset (SOFA 8–12; adjusted HR 0.927; 95 % CI 0.728, 1.181; *p* = 0.540). The estimated survival rates in the very high-risk subset were similar in the treated and untreated groups (SOFA ≥18; adjusted HR 0.915; 95 % CI 0.418, 2.003; *p* = 0.825), but this analysis was not definitive because of the small sample sizes of the subsets.

### Mortality according to other baseline characteristics

**Fig. 5:**
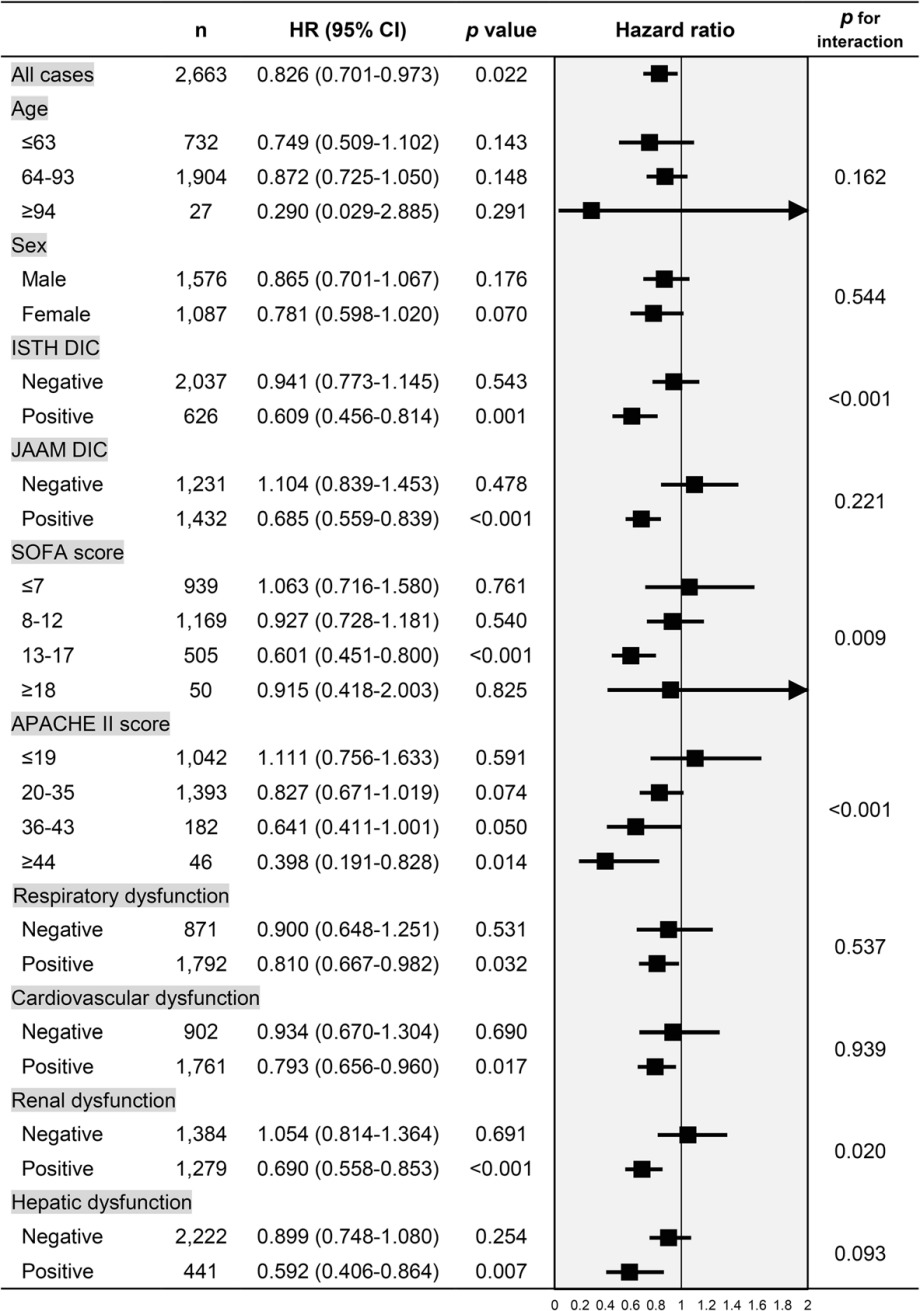
In-hospital mortality across subsets defined according to several baseline characteristics. *HR* hazard ratio, *CI* confidence interval, *ISTH* International Society on Thrombosis and Hemostasis, *DIC* disseminated intravascular coagulation, *JAAM* Japanese Association for Acute Medicine, *SOFA* Sequential Organ Failure Assessment, *APACHE* Acute Physiology and Chronic Health Evaluation

A similar tendency was observed in the analysis of subsets based on a number of other clinical measures of baseline illness severity (Fig. 5 and Additional file 1: Table S6). When the population was separated into subsets according to APACHE II scores, an insignificant reduction in mortality associated with anticoagulant therapy was observed in the moderate-risk and high-risk subsets (APACHE II scores 20–30 and 36–43, respectively), whereas in the low-risk subset (APACHE II score ≤19), there was no difference between the anticoagulant and control groups. In terms of each organ dysfunction, associations between anticoagulant therapy and better outcome were observed only in the subset with organ dysfunction such as respiratory, cardiovascular, renal, and hepatic dysfunction, i.e. only in the population with greater illness severity.

### Adverse events

Bleeding complications stratified by baseline SOFA score subsets are presented in Table 3. Rates of transfusion requirement related to bleeding tended to increase in the treated groups compared with those in the untreated groups in any SOFA subset, but the differences were not statistically significant. Other outcomes, such as occurrence of intracranial haemorrhage and bleeding requiring surgical intervention, were similar in the treated and untreated groups of any SOFA subset.

**Table 3:** Bleeding complications in subsets stratified according to baseline SOFA scores

	Anticoagulant group	Control group	OR (95 % CI)	*P* value
Transfusion related to bleeding				
SOFA score ≤7	36/274 (13 %)	40/665 (6 %)	1.414 (0.817, 2.447)	0.216
SOFA score 8–12	84/615 (14 %)	42/554 (8 %)	1.306 (0.836, 2.041)	0.241
SOFA score 13–17	55/328 (17 %)	14/177 (8 %)	1.739 (0.886, 3.412)	0.108
SOFA score ≥18	8/30 (27 %)	2/20 (10 %)	9.516 (0.861, 105.193)	0.066
Intracranial haemorrhage				
SOFA score ≤7	3/274 (1 %)	1/665 (0.2 %)	6.142 (0.501, 75.288)	0.156
SOFA score 8–12	3/615 (0.5 %)	2/554 (0.4 %)	0.608 (0.080, 4.600)	0.630
SOFA score 13–17	1/328 (0.3 %)	1/177 (0.6 %)	0.286 (0.016, 5.268)	0.400
SOFA score ≥18	0/20 (0 %)	0/30 (0 %)	-	-
Surgical interventions				
SOFA score ≤7	3/274 (1 %)	1/665 (0.2 %)	7.634 (0.642, 90.792)	0.108
SOFA score 8–12	10/615 (2 %)	7/554 (1 %)	1.001 (0.326, 3.074)	0.998
SOFA score 13–17	7/328 (2 %)	3/177 (2 %)	0.824 (0.188, 3.608)	0.797
SOFA score ≥18	1/30 (3 %)	0/20 (0 %)	-	-

Data are expressed as number (percent) or OR (95%CI). *SOFA* Sequential Organ Failure Assessment, *OR* odds ratio, *CI* confidence interval

## Discussion

### Summary of evidence

Numerous large clinical trials of anticoagulant agents did not show a reduction in mortality among all patients with sepsis [8, 9, 11]; thus, it is important to identify specific subsets of patients with sepsis who can benefit from anticoagulant therapy. The present study represents the first attempt to evaluate the benefit profile of anticoagulant therapy in patients with sepsis. The current analyses provide evidence that anticoagulant therapies were associated with lower mortality in the DIC-positive subset, but no such association was evident in the DIC-negative subset. A similar benefit profile was observed in the analysis of the subsets stratified by SOFA score: a significant association between anticoagulant therapy and reduction in mortality was observed in a high-risk subset of patients with sepsis (SOFA score 13–17) but not in the low-risk to moderate-risk subsets (SOFA score ≤12). Furthermore, in patients with sepsis the association between anticoagulant therapy and better outcome was greater in patients with organ dysfunction than in those without organ dysfunction. Overall, the present analyses suggest that anticoagulant therapies may only be effective in sepsis among patients with DIC involving a high risk of death. Thus, future RCTs of anticoagulant therapy for sepsis should focus on specific populations, such as those with sepsis-induced DIC, multiple organ dysfunction, or very severe disease.

### Treatment effects according to baseline DIC status

Our study showing a significant association between anticoagulant therapy and reduced mortality only in the DIC-positive subset agreed with the findings of many observational studies and post hoc subgroup analyses of RCTs [15, 23, 24]. It also showed that anticoagulant therapy is not associated with better outcome when it is administered to patients without DIC. These observations are explained by recent pathophysiological evidence on the innate immune response. Under certain circumstances, thrombosis is considered to play a major physiological role, termed “immunothrombosis”, in immune defence [25–27]. In the non-DIC subset of patients, anticoagulant therapy could have inhibited host-defensive thrombosis, which helps to capture and ensnare pathogens circulating in the blood, and therefore failed to improve mortality. However, in patients with DIC, impairment of the anticoagulant system leads to the overwhelming formation of fibrin and the uncontrolled activation of immunothrombosis, which play a critical role in inducing multiple organ dysfunction syndrome and subsequent death. Therefore, it is reasonable to assume that anticoagulant therapy to inhibit the over-activated coagulation cascade may be useful to improve outcomes in this subset of patients.

### Treatment effects according to baseline disease severity

Our findings on the association between anticoagulant therapy and lower mortality according to disease severity or organ dysfunction agrees with those of many previous studies including a large multicentre RCT of activated protein C or antithrombin [7, 14, 28]. Additionally, our previous meta-analysis of the risk and efficacy of rhTM in sepsis suggests that its efficacy would increase as the risk of death increased [29].

Why were the effects of anticoagulant therapies in this study more evident only when they targeted severely ill patients? One possible reason is that patients who are severely ill are likely to present simultaneously with DIC, which is related to immunothrombosis. Second, differences between groups are likely to be statistically significant if the control event rate is high. Third, anticoagulants such as antithrombin and rhTM have specific anti-inflammatory activities unrelated to anticoagulant activity [30–32], and these anti-inflammatory effects might be clinically evident only in the severely ill patients.

### Adverse events

Bleeding was the most significant adverse event associated with anticoagulant administration. Although the differences were not statistically significant, there was a consistent tendency for bleeding-related transfusions to increase in all of the severity subsets (13–27 % in the anticoagulant group vs. 6–10 % in the control group). The decision to use an anticoagulant depends on the balance between efficacy and the safety of the intervention. Our results showed that anticoagulant therapy may only be useful in the sepsis subset with severe disease because of its beneficial effects on mortality and the relatively low risk of bleeding complications. In contrast, anticoagulant therapy in the overall sepsis population including the low-risk subset should not be recommended because of the risk of bleeding complications and lack of survival benefit.

### Limitations

We acknowledge several limitations of our study. First, it is a non-randomised cohort study and hence suffers from potential selection and ascertainment bias. The indications for treatment and methodology for the treatment intervention being examined were not standardised. The baseline characteristics and intensity of ICU treatments other than anticoagulant therapies were different between the two groups. To cope with these imbalances caused by non-randomisation, we developed a propensity score approach that forces the analysts to explicitly focus on these biases.

Second, the registry data used in the study were retrospectively collected and did not capture detailed information that may be considered as confounding, such as the timing or duration of anticoagulant therapies. We are not confident that biased estimation of the effects can be completely excluded despite robust adjustment with propensity scores.

Third, the present multicentre study did not focus on the treatment effects of specific anticoagulants. Thus, the knowledge obtained from the present study might be inadequate to influence decision making in clinical settings. To verify our hypothesis, we assumed that several of the anticoagulant agents displayed similar effects against sepsis, even though these agents have unique anticoagulant/antiinflammatory mechanisms and pharmacological features. Also, low-dose anticoagulants as prophylaxis against venous thromboembolism were included in both groups. The combination of these three major limitations might cause multiple unmeasured confounders to account for the differences in outcome observed in this study.

Finally, because this study involves subgroup analysis, we cannot deny the potential of accidental false-positive results. The study is also prone to false-negative results due to inadequate power with which to uncover differences in treatment effect, even in the presence of true treatment-effect modification. Further multicentre prospective randomised trials are therefore required to specifically evaluate efficacy and safety.

## Conclusions

Our post hoc subgroup analysis using the multicentre nationwide J-Septic DIC registry in Japan demonstrated an association between anticoagulant therapy and lower mortality only in specific patients with sepsis, who are severely ill and have multiple organ dysfunction, or DIC. Thus, future RCTs of anticoagulant therapy for sepsis should focus on such specific patient populations.

## Key messages

- Patients who will highly benefit from anticoagulant therapy against sepsis require identification
- Effects of anticoagulant therapy were evaluated by baseline disseminated intravascular coagulation status and by stratifying disease severity subsets
- Significant associations between anticoagulant therapy and survival were shown only in specific subsets of sepsis-induced disseminated intravascular coagulation or very severe disease
- Future randomised controlled trials of anticoagulant therapy for sepsis should focus on such specific patient populations

## Abbreviations

APACHE, Acute Physiology And Chronic Health Evaluation; CI, confidence interval; DIC, disseminated intravascular coagulation; HR, hazard ratio; ICUs, intensive care units; ISTH, International Society on Thrombosis and Haemostasis; JAAM, Japanese Association for Acute Medicine; J-septic DIC, Japan Septic Disseminated Intravascular Coagulation; OR, odds ratio; RCT, randomised controlled trial; rhTM, recombinant human soluble thrombomodulin; SOFA, Sequential Organ Failure Assessment; survival CART, classification and regression trees for survival data

## Additional file

Additional file 1 Online supplemental data. (DOCX 63 kb)
