# Supplementary material for: Benefit profile of anticoagulant therapy in sepsis: a nationwide multicentre registry in Japan
Source: Crit Care. 2016 Jul 29;20:229. doi: 10.1186/s13054-016-1415-1 (PMC4966726; doi:10.1186/s13054-016-1415-1)
Supplement: Additional file 1 — Online supplemental data. (DOCX 63 kb) [file 13054_2016_1415_MOESM1_ESM.docx]

**Online Supplemental Data**

| **Table S1** Standard dosage and duration of anticoagulant therapy for treatment of disseminated intravascular coagulation in Japan | | |
| --- | --- | --- |
| Anticoagulant agent | Dosage | Duration |
| Antithrombin | 1500 U/kg/day | 3-5 days |
| Recombinant human soluble thrombomodulin | 380 U/kg/day | 6 days |
| Unfractionated heparin | 120-240 U/kg/day | Not applicable |
| Low-molecular-weight heparin | 75 U/kg/day | Not applicable |
| Danaparoid sodium | 1250 U x 2/day | Not applicable |
| Gabexate mesilate | 20-39 mg/kg/day | Not applicable |
| Nafamostat mesilate | 0.06-0.20 mg/kg/hr | Not applicable |

**Table S2** Ethical approval information of each participating hospital

Osaka General Medical Center (The Institutional Review Board of Osaka General Medical Center, #25-2050), Osaka University Graduate School of Medicine (Ethical Review Board of Osaka University Hospital, #13465 and #13487), Hokkaido University Hospital (The Institutional Review Board of Hokkaido University Hospital, #Ji013-0246), Tohoku University Graduate School of Medicine (Institutional Review Board of Tohoku University School of Medicine, #2013-1-467), Jichi Medical University Saitama Medical Center (Ethics Committee of Jichi Medical University, Saitama Medical Center, #Rin13-98), Shonan Kamakura General Hospital (The Institutional Review Board of Shonan Kamakura General Hospital, #Syokama20140220-1), Jikei University School of Medicine (The Ethics Committee of the Jikei University School of Medicine for Biomedical Research, #25-315 7450), University of Occupational and Environmental Health (Ethics Committee of Medical Research, University of Occupational and Environmental Health, #H26-040), Nihon University School of Medicine (Research Review Board of Nihon University School of Medicine, Itabashi Hospital, #RK-140411-7), Ohta General Hospital Foundation Ohta Nishinouchi Hospital (The Institutional Review Board of Ohta Nishinouchi Hospital, no specific identification number), JA Hiroshima General Hospital (The Institutional Review Board of JA Hiroshima General Hospital, #14-6), Saitama Red Cross Hospital (Hospital Ethical Committee of Saitama Red Cross, #20140514-2), Wakayama Medical University (The Ethical Review Board of Wakayama Medical University, #1365), Japan Red Cross Maebashi Hospital (Research Review Board of Japan Red Cross Maebashi Hospital, #25-36), Kyushu University Hospital (Kyushu University Institutional Review Board for Clinical Research, #26-146), Fukuoka University Hospital (Institutional Review Board of Fukuoka University Hospital, #14-4-15), Ibaraki Prefectural Central Hospital (Clinical Research Ethics Review Committee of Ibaraki Prefectural Central Hospital, #25-79), Nagasaki University Hospital (the Institutional Review Board of Nagasaki University Hospital, #14012754), Tokyo Medical University Hachioji Medical Center (The Institutional Review Board of Tokyo Medical University Hachioji Medical Center, #H-13), Kyoto First Red Cross Hospital (The Ethical Committee of Kyoto Daiichi Red Cross Hospital, #362), Saiseikai Yokohama Eastern Hospital (Saiseikai Yokohama Eastern Hospital Ethics Committee, #2013065), Asahikawa Medical University (Asahikawa Medical University Research Ethics Committee, #1737), Nippon Medical School Chiba Hokusoh Hospital (The Ethical Review Board of Nippon Medical School Chiba Hokusoh Hospital, #409), Kameda Medical Center (Kameda Medical Center, Research Ethics Committee, #13-089), Asahikawa Red Cross Hospital (Clinical Research Ethics Review Committee of Asahikawa Redcross Hospital, #201336-2), Graduate School of Medicine, University of the Ryukyus (The Ethical Committee of the University of Ryukyus, #208), Gifu University Hospital (Medical Review Board of Gifu University Graduate School of Medicine, #26-30), Saga University Hospital (Ethics Committee , Faculty of Medicine , Saga University, #26-6), Steel Memorial Muroran Hospital (The Ethical Committee of Steel Memorial Murrain Hospital, no specific identification number), Sapporo City General Hospital (The Ethical Committee of Sapporo City General Hospital, #H25-047-191), Ehime University Hospital (the Institutional Review Board of Ehime University Hospital, #Aidaiibyorin1402011), Tomishiro Central Hospital (The Institutional Review Board of Tomishiro Central Hospital, #H26R002), Akashi City Hospital (The Institutional Review Board of Akashi City Hospital, # 2014-001), Sendai City Hospital (The Ethical Committee of Sendai city hospital, #Senbyoso595), Hakodate Municipal Hospital (Ethical Review Board of Hakodate Municipal Hospital, no specific identification number), Mie University Hospital (the Clinical Research Ethics Review Committee of Mie University Hospital, #2740), Gunma University (Institutional Review Board of Gunma University Hospital, no specific identification number), KKR Sapporo Medical Center (Ethical Review Board of KKR Sapporo Medical Center, #25-25), Seirei Mikatahara General Hospital (The Ethical Committee of Seirei Mikatahara General Hospital, #13-27), Hyogo College of Medicine (The Ethics Review Board of Hyogo College of Medicine, #1681)

| **Table S3** Diagnostic scores for disseminated intravascular coagulation | | |
| --- | --- | --- |
|  | ISTH criteria | JAAM criteria |
| Underlying disorder known to be associated with DIC | Required | Required |
| SIRS score | - | 0-2: 0 points  ≥3: 1 point |
| Platelet count | >10 x 10^4^/μL: 0 points  ≤10 x 10^4^/μL: 1 point  ≤5 x 10^4^/μL: 2 points | ≥12 x 10^4^/μL: 0 points  8-12 x 10^4^/μL (or >30% decrease/24 hrs): 1 point  <8 x 10^4^/μL (or >50% decrease/24 hrs): 3 points |
| Prothrombin time | <3 sec: 0 points  3-6 sec: 1 point  ≥6 sec: 2 points | Prothrombin time ratio:  <1.2: 0 points  ≥1.2: 1 point |
| Fibrinogen | >100 mg/dL: 0 points  ≤100 mg/dL: 1 point | - |
| Fibrin/fibrinogen degradation products | No increase: 0 points  Moderate increase: 2 points  Marked increase: 3 points | <10 μg/mL: 0 points  10-25 μg/mL: 1 point  ≥25 μg/mL: 3 points |
| Total DIC score | No DIC: <5 points  DIC: ≥5 points | No DIC: <4 points  DIC: ≥4 points |
| *ISTH* International Society on Thrombosis and Haemostasis, *JAAM* Japanese Association for Acute Medicine, *DIC* disseminated intravascular coagulation, *SIRS* systemic inflammatory response syndrome | | |

| **Table S4** The 32 variables used to calculate propensity scores in the logistic regression model | |
| --- | --- |
| Patient characteristics | 1) age, 2) sex |
| Illness severity | 3) SIRS score, 4) SOFA score, 5) APACHE II score, 6) JAAM DIC score, 7) ISTH overt DIC score, 8) positive blood culture |
| 9) Source of ICU admission | Emergency department/ward/other hospital |
| Pre-existing condition | 10) liver insufficiency, 11) chronic heart failure, 12) chronic respiratory disorder, 13) chronic haemodialysis, 14) immunocompromised |
| New organ dysfunction | 15) respiratory, 16) cardiovascular, 17) renal, 18) hepatic, 19) coagulation |
| 20) ICU characteristics | Closed ICU/open ICU/other |
| 21) Primary source of infection | Abdomen/lung/urinary tract/bone+soft tissue/central nervous system/other+unknown |
| 22) Causal microorganisms | Gram-positive bacteria/Gram-negative bacteria/mixed organisms/other/unknown |
| Anticoagulant therapy not for DIC | 23) nafamostat mesilate for renal replacement therapy, 24) heparin for venous thromboembolism prophylaxis, 25) warfarin, 26) anti-platelet drugs, 27) others |
| Other therapeutic interventions | 28) immunoglobulin, 29) low-dose steroid, 30) renal replacement therapy, 31) PMX-DHP, 32) surgical intervention |
| *SIRS* Systemic Inflammatory Response Syndrome, *SOFA* Sequential Organ Failure Assessment, *APACHE* Acute Physiology and Chronic Health Evaluation, *JAAM* Japanese Association for Acute Medicine, *ISTH* International Society on Thrombosis and Hemostasis, *DIC* disseminated intravascular coagulation, *ICU* intensive care unit, *PMX-DHP* polymyxin B direct haemoperfusion | |

| **Table S5** Baseline characteristics of patients in the high-risk subset (SOFA score 13-17) treated or untreated with anticoagulant | | | | |
| --- | --- | --- | --- | --- |
|  | Overall  (n = 505) | Anticoagulant  group (n = 328) | Control group  (n = 177) | *p* value |
| Patient characteristics |  |  |  |  |
| Age in years | 71 (62-79) | 70 (62-79) | 71 (62-80) | 0.867 |
| Male sex | 305 (60%) | 196 (60%) | 109 (62%) | 0.689 |
| Illness severity |  |  |  |  |
| SIRS score | 3 (3-4) | 3 (3-4) | 3 (3-4) | 0.699 |
| SOFA score | 14 (13-15) | 14 (13-15) | 14 (13-15) | 0.811 |
| APACHE II score | 29 (25-36) | 29 (25-35) | 31 (25-38) | 0.015 |
| ISTH DIC score | 4 (3-6) | 5 (3-6) | 4 (3-5) | 0.011 |
| JAAM DIC score | 6 (4-7) | 6 (4-8) | 5 (3-6) | 0.001 |
| Source of ICU admission |  |  |  | 0.558 |
| Emergency department | 218 (43%) | 136 (42%) | 82 (46%) | - |
| Ward | 154 (31%) | 104 (32%) | 50 (28%) | - |
| Other hospital | 133 (26%) | 88 (27%) | 45 (25%) | - |
| Pre-existing condition |  |  |  |  |
| Liver insufficiency | 8 (2%) | 4 (1%) | 4 (2%) | 0.372 |
| Chronic heart failure | 33 (7%) | 28 (9%) | 5 (3%) | 0.013 |
| Chronic respiratory disorder | 20 (4%) | 10 (3%) | 10 (6%) | 0.153 |
| Chronic haemodialysis | 55 (11%) | 34 (10%) | 21 (12%) | 0.606 |
| Immunocompromised | 73 (15%) | 48 (15%) | 25 (14%) | 0.876 |
| New organ dysfunction (SOFA subscores ≥2) | |  |  |  |
| Respiratory | 444 (88%) | 288 (88%) | 156 (88%) | 0.913 |
| Cardiovascular | 485 (96%) | 317 (97%) | 168 (95%) | 0.341 |
| Renal | 417 (83%) | 275 (84%) | 142 (80%) | 0.307 |
| Hepatic | 144 (29%) | 94 (29%) | 50 (28%) | 0.922 |
| Coagulation | 345 (68%) | 229 (70%) | 116 (66%) | 0.324 |
| Primary source of infection |  |  |  | 0.687 |
| Abdomen | 168 (33%) | 113 (35%) | 55 (31%) | - |
| Lung | 121 (24%) | 74 (23%) | 47 (27%) | - |
| Urinary tract | 88 (17%) | 61 (19%) | 27 (15%) | - |
| Bone/soft tissue | 49 (10%) | 33 (10%) | 16 (9%) | - |
| Central nervous system | 13 (3%) | 8 (2%) | 5 (3%) | - |
| Other/unknown | 66 (13%) | 39 (12%) | 27 (15%) | - |
| Other therapeutic interventions |  |  |  |  |
| Immunoglobulin | 204 (40%) | 167 (51%) | 37 (21%) | <0.001 |
| Low-dose steroid | 195 (39%) | 141 (43%) | 54 (31%) | 0.006 |
| Renal replacement therapy | 310 (61%) | 236 (72%) | 74 (42%) | <0.001 |
| PMX-DHP | 179 (35%) | 144 (44%) | 35 (20%) | <0.001 |
| Surgical intervention | 212 (42%) | 159 (49%) | 53 (30%) | <0.001 |
| Data are expressed as group medians (interquartile range) or proportion (%)  *SIRS* Systemic Inflammatory Response Syndrome, *SOFA* Sequential Organ Failure Assessment, *APACHE* Acute Physiology and Chronic Health Evaluation, *ISTH* International Society on Thrombosis and Hemostasis, *DIC* disseminated intravascular coagulation, *JAAM* Japanese Association for Acute Medicine, *ICU* intensive care unit, *PMX-DHP* polymyxin B direct haemoperfusion | | | | |

| **Table S6** In-hospital mortality across subsets defined according to primary source of infection and volume of sepsis patients at each institution | | | | |
| --- | --- | --- | --- | --- |
|  | n | HR (95% CI) | *p* value | *p* for interaction |
| Primary source of infection |  |  |  | 0.269 |
| Abdomen | 881 | 0.770 (0.571-1.039) | 0.087 |  |
| Lung | 677 | 1.192 (0.890-1.597) | 0.238 |  |
| Urinary tract | 456 | 0.426 (0.243-0.747) | 0.003 |  |
| Bone/soft tissue | 309 | 0.711 (0.436-1.157) | 0.169 |  |
| Central nervous system | 57 | 0.813 (0.313-2.112) | 0.671 |  |
| Other/unknown | 283 | 0.793 (0.504-1.247) | 0.315 |  |
| Patient volume^a^ |  |  |  | 0.591 |
| Low-volume (33 ICUs) | 1,569 | 0.935 (0.744-1.177) | 0.569 |  |
| High-volume (9 ICUs) | 1,144 | 0.762 (0.599-0.971) | 0.028 |  |
| ^a^All ICUs were divided into low-volume (<99 included patients) and high-volume (≥100 patients).  *HR* hazard ratio, *CI* confidence interval, *ICU* intensive care unit | | | | |

The point estimate of hazard ratio for death in each subset is indicated

by a solid square and the 95% confidence interval (CI) by the horizontal lines.
